# Characterization of Sialic Acid-Binding Immunoglobulin-Type Lectins in Fish Reveals Teleost-Specific Structures and Expression Patterns

**DOI:** 10.3390/cells9040836

**Published:** 2020-03-31

**Authors:** Kim F. Bornhöfft, Joan Martorell Ribera, Torsten Viergutz, Marzia T. Venuto, Ulrike Gimsa, Sebastian P. Galuska, Alexander Rebl

**Affiliations:** 1 Institute of Reproductive Biology, Leibniz Institute for Farm Animal Biology (FBN), Wilhelm-Stahl-Allee 2, 18196 Dummerstorf, Germany; bornhoefft@fbn-dummerstorf.de (K.F.B.); viergutz@fbn-dummerstorf.de (T.V.); venuto@fbn-dummerstorf.de (M.T.V.); 2 Institute of Genome Biology, FBN, Wilhelm-Stahl-Allee 2, 18196 Dummerstorf, Germany; martorell-ribera@fbn-dummerstorf.de; 3 Institute of Behavioural Physiology, FBN, Wilhelm-Stahl-Allee 2, 18196 Dummerstorf, Germany; gimsa@fbn-dummerstorf.de

**Keywords:** acute stress, ITIM, Salmonidae, siglecs, sialic acids, vertebrate evolution

## Abstract

The cellular glycocalyx of vertebrates is frequently decorated with sialic acid residues. These sialylated structures are recognized by sialic acid-binding immunoglobulin-type lectins (Siglecs) of immune cells, which modulate their responsiveness. Fifteen Siglecs are known to be expressed in humans, but only four Siglecs are regularly present in fish: Siglec1, CD22, myelin-associated glycoprotein (MAG), and Siglec15. While several studies have dealt with the physiological roles of these four Siglecs in mammals, little is known about Siglecs in fish. In the present manuscript, the expression landscapes of these Siglecs were determined in the two salmonid species *Oncorhynchus mykiss* and *Coregonus maraena* and in the percid fish *Sander lucioperca.* This gene-expression profiling revealed that the expression of *MAG* is not restricted to neuronal cells but is detectable in all analyzed blood cells, including erythrocytes. The teleostean MAG contains the inhibitory motif ITIM; therefore, an additional immunomodulatory function of MAG is likely to be present in fish. Besides *MAG*, *Siglec1*, *CD22*, and *Siglec15* were also expressed in all analyzed blood cell populations. Interestingly, the expression profiles of genes encoding Siglecs and particular associated enzymes changed in a gene- and tissue-specific manner when *Coregonus maraena* was exposed to handling stress. Thus, the obtained data indicate once more that stress directly affects immune-associated processes.

## 1. Introduction

Innate immunity is of even more paramount importance for fish health than adaptive immune mechanisms [1]. More than 50 regulators of innate immunity are known in fish today [2]; these regulators maintain the balance between pathogen defense and pathophysiological manifestations. Some of these innate-immune regulators recognize self-associated molecular patterns (SAMPs), a heterogeneous group of molecules that stimulate inhibitory receptors to dampen immune responses [3,4,5]. The family of sialic acids consists of more than 50 members [6] and belongs to the group of SAMPs [7]. Sialic acids are frequently located at the terminal ends of glycans on glycoproteins and the glycolipids that coat all mammalian cells in a glycocalyx. Immune cells abundantly express sialic acid-binding immunoglobulin-type lectins (Siglecs), which interact with these sialylated glycoconjugates and thus prevent an excessive immunological response [8,9].

The Siglecs are divided into two subgroups: the CD33-related Siglecs and the highly conserved Siglecs, including Siglec1 (sialoadhesin), CD22 (Siglec2), MAG (Siglec4), and Siglec15. Both subgroups comprise Siglecs that suppress the immune response or activate the immune system [9,10]. The inhibitory function of Siglecs is mediated by an intracellular immune-receptor tyrosine-based inhibition motif (ITIM; signature: [I/V/L/S]-X-Y-X-X-[L/V] [9]), which is absent in immune-activating Siglecs (Siglec14, Siglec15, and Siglec16 in humans). These Siglec receptors interact with DNAX proteins (DAP10/DAP12), which carry immune-receptor tyrosine-based activation motifs (ITAMs) [9,11]. To date, fifteen and nine Siglecs have been identified in human and mouse, respectively, each expressed by defined cell types and responding to a particular set of ligands [12,13,14]. Remarkably, Siglec1, CD22, MAG, and Siglec15 were already present in the animal kingdom before the separation of tetrapods and teleost fishes more than 400 million years ago [15]. However, little is known about the physiological role of Siglecs in teleosts [16,17] including their tissue-specific basal expression profiles. For this reason, the present study aimed at the analysis of the expression landscapes of Siglec1, CD22, MAG, and Siglec15 in teleost fish. The expression profiling was complemented by in-depth analyses of the structures of the aforementioned Siglec receptors. These investigations focused on three aquaculture fish species of economic importance, (i) pikeperch (*Sander lucioperca*) as a representative of diploid percid fish, (ii) rainbow trout (*Oncorhynchus mykiss*) and (iii) maraena whitefish (*Coregonus maraena*) as representatives of the pseudotetraploid salmonid fishes that are currently undergoing species-specific reploidization processes [18]. Notably, the rainbow trout has been adapted to intensive farming for decades, whereas maraena whitefish is a novel aquaculture species and a useful model for dissecting the response of a pseudo-wild fish to anthropogenic environments. Our previous transcriptomic analyses on the stress physiology of the maraena whitefish revealed that the level of MAG transcripts is modulated by a factor of approximately two in different organs after exposure to temperature and stocking-density stress [19,20]. These findings suggested that Siglecs could potentially be biomarkers for immune and stress responses in teleost fish. To address whether stress affects the expression of Siglec-encoding genes, maraena whitefish were exposed to handling procedures [19,20]. The presently outlined study provides novel insights into the physiological roles of Siglecs in teleost fish.

## 2. Materials and Methods

### 2.1. Fish Husbandry and Experimental Treatment

The animals used for this study were provided by the Institute for Fisheries of the State Research Centre for Agriculture and Fishery Mecklenburg–Western Pomerania and BiMES Binnenfischerei GmbH (Friedrichsruhe, Germany). Fish were held in freshwater recirculation systems under a 12:12 day-and-night cycle at 18 °C. Water quality was maintained by automated purification and disinfection (bio-filter and UV light). In addition, the concentrations of NH_4_^+^, NO_2_^−^, NO_3_^−^, and NH_3_ in the water, pH value, temperature, and oxygen saturation were constantly recorded. The feeding material consisted of commercial dry pellets (4.5 mm, INICIO Plus; BioMar, Brande, Denmark), which were distributed by automatic feeders at a daily rate of 0.8–4.0%, depending on the biomass of the fish.

Maraena whitefish were acclimatized in the experimental tanks connected to a recirculation system for a period of at least three weeks. Acute handling stress was induced by chasing, netting and the transfer of fish to another tank for a period of one minute. Eight stressed and eight control fish were euthanized with an overdose of 2-phenoxyethanol (0.7 mL/L; Sigma-Aldrich/Merck, Munich, Germany) and then underwent spine sectioning at the skull level. These procedures followed the standards described in the German Animal Welfare Act (§ 4(3) TierSchG) and were approved by the Landesamt für Landwirtschaft, Lebensmittelsicherheit und Fischerei (Mecklenburg–Vorpommern, Germany; approval ID: LALLF M-V/TSD/7221.3-1-069/18; date of approval: January 16, 2019). The brain of each maraena whitefish was dissected into the hypothalamus, telencephalon, and hindbrain and sampled together with the spleen, liver, white muscle, gills, head kidney (HK), and heart. Samples were snap-frozen in liquid nitrogen and stored at −80 °C. Husbandry and sampling of pikeperch and rainbow trout have been described elsewhere [21,22].

### 2.2. Isolation and Microscopic Characterization of Head Kidney Cells from Maraena Whitefish

For HK cell isolation and cell sorting, HKs from freshly slaughtered maraena whitefish were dissected and put into a Dulbecco’s Modified Eagle Medium (DMEM; Gibco/Thermo Fisher Scientific, Bremen, Germany). The tissue was homogenized using a steel sieve (500 µm, Carl Roth, Karlsruhe, Germany) and then filtered through two cell strainers (200 µm, pluriSelect Life Science, Leipzig, Germany; 100 µm, Falcon/Fisher Scientific, Schwerte, Germany). After centrifugation (524× *g*, 5 min) and resuspension in 3 mL DMEM, the cell suspension was added onto an isotonic Percoll (Sigma-Aldrich/Merck) gradient (3 mL, *ρ* = 1.084 g/mL) and centrifuged at 800× *g* for 30 min at 6 °C with minimum deceleration. The erythrocyte pellet was stored at −80 °C for further RNA extraction, while the cell band at the interface was collected in the DMEM and the volume was adjusted for cell counting. Cell number and cell viability were determined using the Cellometer Auto 2000 (Nexcelom Bioscience, Lawrence, MA, USA). In addition, a portion of the cells separated by the Percoll gradient were placed on glass slides, stained with a May-Grünwald-Giemsa solution (Brand, Wertheim, Germany; Carl Roth), and then microscopically observed using a Nikon TMS-F microscope and a Nikon Coolpix E5000 camera with an MDC Lens (Nikon, Tokyo, Japan).

### 2.3. Flow Cytometry

Flow cytometry was performed using a MoFlo XDP high-speed cell sorter (Beckman Coulter, Krefeld, Germany) with an incorporated, air-cooled sapphire laser (488 nm, 100 mW). A total of ~20 million HK cells were sorted through a 70-µm nozzle at 60 psi on purify mode into two fractions, low side-scattering intensity (fraction I) and high side-scattering intensity (fraction II). Fractions I and II were collected in phosphate-buffered saline (PBS), centrifuged at 500× *g* for 5 min, and used for RNA extraction. Subsequently, cell type-specific gene expressions were profiled, as described in detail in [23].

### 2.4. RNA Isolation

Approximately 50 µg of each of the individual tissue samples were placed in separate reaction tubes containing 1 mL of TRIzol Reagent (Life Technologies/Thermo Fisher Scientific) and homogenized using the Precellys24 Homogeniser (6000 rpm, 30 s). After the addition of chloroform and a centrifugation step (12,000× g, 15 min, 4 °C), the RNA contained in the resulting aqueous phase was purified using the RNeasy Mini Kit (Qiagen, Hilden, Germany). RNA was isolated from cells and purified using the Isolate 2 RNA Micro Kit (Bioline, Luckenwalde, Germany) according to the manufacturer’s instructions and without a previous treatment with TRIzol Reagent. The quality of the purified RNA was checked using horizontal agarose-gel electrophoresis. RNA concentration was determined using the NanoDrop 1000 spectrophotometer (Thermo Fisher Scientific).

### 2.5. Primer Design and Quantitative PCR

Species-specific quantitative PCR (qPCR) primers were designed for the target genes using PSQ Assay Design 1.0.6 software (Biotage AB, Uppsala, Sweden). Amplicon length ranged from 140 to 180 bp (Table S1). Coding sequences for rainbow trout were retrieved from the NCBI public database. To identify Siglec sequences from pikeperch or maraena whitefish, we aligned the orthologous sequences from yellow perch (*Perca flavescens*) or rainbow trout, Atlantic salmon (*Salmo salar*) and coho salmon (*Oncorhynchus kisutch*), respectively, with the recently published pikeperch genome [24] or our RNA-seq read collection from maraena whitefish [25] using Bowtie 2 software (v. 2.2.4; http://bowtie-bio.sourceforge.net/bowtie2/index.shtml). These alignments were then indexed and sorted with the Samtools software package (v. 16; http://www.htslib.org/), and final consensus sequences were obtained with Ugene software (v. 1.29; http://ugene.net/). The amplicon sequences selected from pikeperch were highly identical to the counterpart sequences selected from yellow perch (*SIGLEC1*: 92%; *CD22*: 98%; *MAG*: 91%), but they shared only moderate levels of identity with the respective sequences of other fish species. Moreover, we did not find any *SIGLEC15*/*CD33L3* sequences from percid fish species in the public databases, so we used a *CD33* sequence from barred knifejaw (*Oplegnathus fasciatus*) instead. To take the uncertain assignment of the aforementioned sequences into account, we extended the gene names of the pikeperch sequences by ‘-like’ (abbreviated as ‘L’).

The integrity and specificity of the PCR products were assessed via standard PCRs (HotStarTaq Plus DNA Polymerase, Qiagen) and single qPCR analyses (LightCycler 480 System, Roche, Mannheim, Germany; SensiFAST SYBR No-ROX Kit, Bioline). A multiplex qPCR analysis was performed with the 48.48 Dynamic Array IFC chip (Fluidigm, South San Francisco, CA, USA) and the BioMark HD-System (Fluidigm) using the nucleotide-binding EvaGreen fluorescence dye (Bio-Rad, Feldkirchen, Germany). In detail, 1 µL of the extracted RNA was reverse-transcribed with the Reverse Transcription Master Mix (Fluidigm). Then, the designed primer pairs and the PreAmp Master Mix (Fluidigm; 100 µM of mix per pair) were used to perform 11 pre-amplification cycles with the individual cDNA samples adjusted at 10 ng/5 μL. The pre-amplified products were treated with exonuclease I (ExoI; New England BioLabs, Frankfurt am Main, Germany) and subsequently diluted in a pre-mixed solution of SsoFast EvaGreen Supermix with Low ROX (Bio-Rad) and 20 × DNA binding dye sample loading reagent. The sample and primer mixes were transferred to the respective inlets of the 48.48 Dynamic Array IFC chip, which was thereafter primed in the BioMark IFC Controller MX (Fluidigm), running the Load Mix 48.48 GE script. The loaded array chip was then placed in the BioMark HD-System (Fluidigm) to proceed with the qPCR according to the GE 48 × 48 Fast PCR+Melt v2.pcl cycling program. Fluidigm RealTime PCR Analysis Software (v. 3.0.2, https://www.fluidigm.com/software) was used to analyze the qPCR results. To obtain the relative copy number of each transcript, a serial dilution-based standard curve (10^2^–10^6^ copies) was used and the copy number was normalized with the geometric mean of three suitable reference genes (*EEF1A1b*, *RPL9*, and *RLP32* for maraena whitefish; *EEF1A1*, *ACTB*, and *RPS5* for rainbow trout; and *EEF1A1*, *RPS5*, and *RLP32* for pikeperch) [21,26,27,28].

### 2.6. Cloning

Since we retrieved only gene fragments of *CD22* and *MAG* from our transcriptome of maraena whitefish, we derived primers from the 5′ and 3′ ends of the respective open reading frames. First, a SuperScript II Reverse Transcriptase Kit (Invitrogen/Thermo Fisher Scientific) was used to transcribe a total of 1 µg of RNA into cDNA. This reverse transcription was carried out at 42 °C for 50 min, followed by an inactivation step at 70 °C for 15 min. Purification of the cDNA was performed using a High Pure PCR Product Purification Kit (Roche), and the resulting cDNA was diluted in 100 µL of distilled water. Subsequently, we used the HotStarTaq Plus DNA Polymerase (Qiagen) to generate the PCR products of the full-length open reading frames. The purified (High Pure PCR Product Purification Kit; Roche) amplicons were inserted into a pGEM-T-Easy vector (Promega, Walldorf, Germany). The obtained plasmids were sequenced using the universal SP6/T7 primers and a MegaBACE capillary sequencer (GE Healthcare, Freiburg im Breisgau, Germany). Twelve clones were picked and analyzed per amplified sequence fragment.

### 2.7. In Silico Analyses

Sequence alignments were performed using the Clustal Omega tool of EMBL-EBI (https://www.ebi.ac.uk/Tools/msa/clustalo/). The sequences of CD22, MAG and Siglec15 were derived by blasting the orthologous sequences of rainbow trout (*O. mykiss*; Om), coho salmon (*O. kisutch;* Ok), Atlantic salmon (*S. salar*; Ss), yellow perch (*P. flavescens*; Pf), and barred knifejaw (*O. fasciatus*; Of) against the sequence assemblies of maraena whitefish and pikeperch [24,29]. In addition, the CD22 and MAG sequences of maraena whitefish were completed by sequencing the results.

We retrieved the following sequences from the NCBI database: *Danio rerio* (zebrafish) MAG: XP_021337068; *Takifugu rubripes* (pufferfish) MAG: XP_011616490; *Mus musculus* (mouse) Siglec15: NP_001094508, MAG: XP_030098048; CD22: NP_033975; and *Homo sapiens* (human) Siglec15: NP_998767, MAG: AAB58805, CD22: NP_001762. The positions of the transmembrane domains of murine and human Siglec15 were retrieved from Uniprot; the transmembrane areas of Siglec15 from maraena whitefish and rainbow trout were predicted using SMART (http://smart.embl-heidelberg.de/). The V-set, Ig-like domains of murine and human Siglec15 were determined using the SMART program; the V-set, Ig-like domains of MAG and Siglec15 from fishes were estimated based on sequence alignments; and the V-set, Ig-like domains of human MAG and murine MAG (pdb sequence: 5LF5) were determined using the SMART program. In addition, the V-set, Ig-like domain of human and mouse CD22 was determined using the SMART program, while the V-set, Ig-like domains of CD22 from maraena whitefish and rainbow trout were defined by sequence alignment.

The 3D modelling of the sialic acid-binding domain (V-set, Ig-like domain) of CD22 from human and maraena whitefish was performed using YASARA 19.9.17. The structure of human CD22 was given by the pdb sequence: 5VKM, published by Ereño-Orbea et al. [30]. Based on Uniprot, the sequence was shortened to the first Ig-domain, responsible for sialic acid recognition. The sequence of the V-set domain of CD22 from maraena whitefish was obtained by sequence alignment with the sequence of the V-set, Ig-like domain of human CD22.The 3D-modelling of the sialic acid-binding domain (V-set, Ig-like domain) of MAG from mouse and maraena whitefish was also performed using YASARA 19.9.17. The structure of the murine MAG was determined by Pronker et al. [31]. Using the generated alignments, the sequence of murine MAG was shortened to the first Ig domain, which is responsible for sialic acid-binding. The sequence of the V-set, Ig-like domain of MAG from maraena whitefish was based on our cloned sequences.

The mechanistic interaction of Siglecs and associated factors was illustrated using the Ingenuity Pathways Analysis software (IPA; Qiagen Bioinformatics software solutions) based on the Ingenuity Knowledge Base. The constructed informal diagram was manually edited using the Path Designer tool (IPA).

## 3. Results and Discussion

### 3.1. The Expression of Siglec-Encoding Genes in Different Tissues of Salmonid and Percid Fishes

The expression of Siglec-encoding genes in different lymphoid and non-lymphoid tissues of the economically important farm-fish families *Salmonidae* and *Percidae* has, to our knowledge, not yet been described in detail. To pave the road for future immunological studies of the sialic acid-dependent regulation of immune processes in bony fish, we performed structural analyses and multiplex qPCR measurements of the piscine Siglecs *Siglec1*, *CD22*, *MAG*, and *Siglec15*.

**Figure 1:**
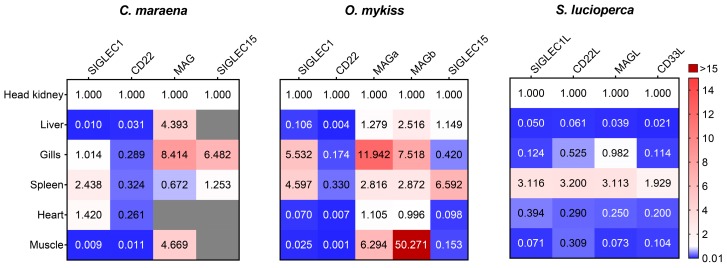
Tissue-specific expression of *Siglec1*, *CD22*, *MAG*, and *Siglec15*/*CD33L* in maraena whitefish (*C. maraena*), rainbow trout (*O. mykiss*), and pikeperch (*S. lucioperca*). The qPCR data were normalized by three reference genes. The resulting transcript numbers from the head kidney (HK) were set at 1.0, and the transcript numbers of the same gene in all other tissues were expressed as fractions. Lower and higher transcript values than those of the HK are highlighted in blue and red, respectively, according to the given color code. Non-detectable transcript numbers are indicated by gray fields.

Siglec1 plays an indispensable role in innate and humoral immunity, even though it contains no immunomodulatory motifs [32]. The expression analyses (Figure 1) demonstrated that *Siglec1* mRNA was most abundant in the spleens and HKs of pikeperch (>2 × 10^7^ copies/µg RNA) and in the spleens and gills of maraena whitefish (>2 × 10^3^ copies/µg RNA) and rainbow trout (>3 × 10^3^ copies/µg RNA). The teleostean HK is considered the functional counterpart of the mammalian bone marrow [1]. It contains considerable amounts of lymphocytes and macrophages [33], similar to the spleen and gills. Probably, *Siglec1* is mainly expressed on those immune cells, since it has been reported that mammalian *Siglec1* is highly expressed on splenic and lymph-node macrophages [9,13].

CD22 was assigned as an activation marker for mature B cells in mammals, and the interaction of CD22 with the B-cell receptor (BCR) has been well established in mammals. The highest levels of *CD22* were found in the HKs of the salmonids maraena whitefish and rainbow trout (>2 × 10^5^ copies/µg RNA) as well as in the HKs of pikeperch (>3 × 10^6^ copies/µg RNA). The high *CD22* copy number might indicate an analogous interaction between the CD22 and B cells of the teleostean HK.

Siglec15 is an immune-activating Siglec interacting with DNAX proteins [9,11]. The salmonid fish species shared high expression levels of *Siglec15* in the spleen (>15 × 10^3^ copies/µg RNA). This observation was in line with the fact that mammalian macrophages are the dominant expression site of *Siglec15* [13,34].

MAG is mainly involved in the stabilization of axon-myelin interactions, the inhibition of neurite growth, and the inhibition of axon regeneration in mammals [35]. The copy numbers of *MAG* were present in a range of tissues across the three analyzed fishes, with the highest *MAG* levels in the gills (>8 × 10^3^ copies/µg RNA). Notably, two *MAG* ohnologs were expressed in rainbow trout: *MAGa* and *MAGb*, which are located on chromosomes 2 and 3, respectively. *MAGa* showed at least twice as many transcript numbers as *MAGb* in the analyzed tissues, with muscle containing the highest number of *MAGb* transcripts (>2 × 10^3^ copies/µg RNA).

**Figure 2:**
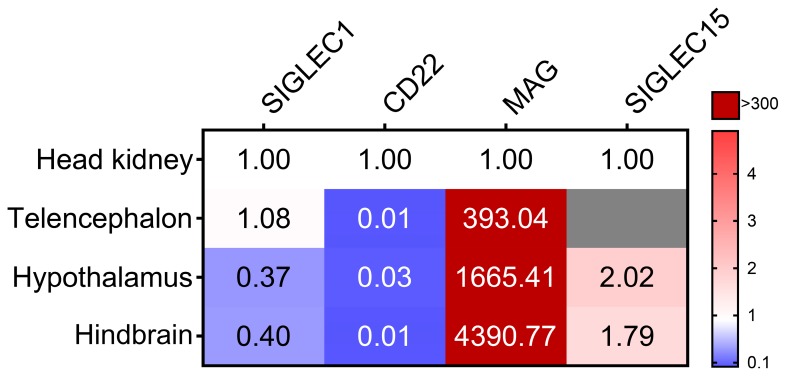
Expression of *Siglec1*, *CD22*, *MAG*, and *Siglec15* in the different brain regions of maraena whitefish. The qPCR data were normalized by the reference genes *RPL9*, *EEF1A1b*, and *RPL32*. The resulting transcript numbers of the same genes in different brain regions are shown relative to the respective transcript levels in the HKs, which were set at 1.0. Transcript values compared with those found in HKs are colored according to the code on the right. Non-detectable transcript numbers are indicated by a gray field.

Since in mammals the highest amounts of MAG can be found in the central nervous system [35], we also inspected the expression of *MAG* together with that of *SIGLEC1*, *CD22*, and *SIGLEC15* in different regions of the brains (hypothalami, telencephalons, and hindbrains) of maraena whitefish (Figure 2). While the transcripts of *Siglec1*, *CD22*, and *Siglec15* were detected at low or moderate levels (<2.5 × 10^3^ copies/µg RNA), we detected extremely high levels (~2.6 × 10^7^ copies/µg RNA) of *MAG* in the brains of maraena whitefish, especially in the hindbrains (>5 × 10^7^ copies/µg RNA). The *MAG* levels in the hindbrains exceeded even the relatively high *MAG* copy numbers in the gills, muscles, and livers of maraena whitefish by 300- to 4000-fold (Figure 1 and Figure 2).

The expression of *Siglec15* was absent in telencephalon, but comparably high in the hypothalamus of maraena whitefish (~2.5 × 10^3^ copies/µg RNA). The murine SiglecH has previously been described as activating an immune response in microglia cells [36]. SiglecH is known to interact with DAP12 and enhance the phagocytotic activity of glioma cells in mice [36,37]. It is conceivable that Siglec15 may play a similar immune-regulatory function in fish brains. This also applies to CD22, which is expressed in mammalian microglia cells to decrease inflammatory effects [38].

### 3.2. The Expression Patterns of Siglec-Encoding Genes in Cell Populations of Maraena Whitefish

**Figure 3:**
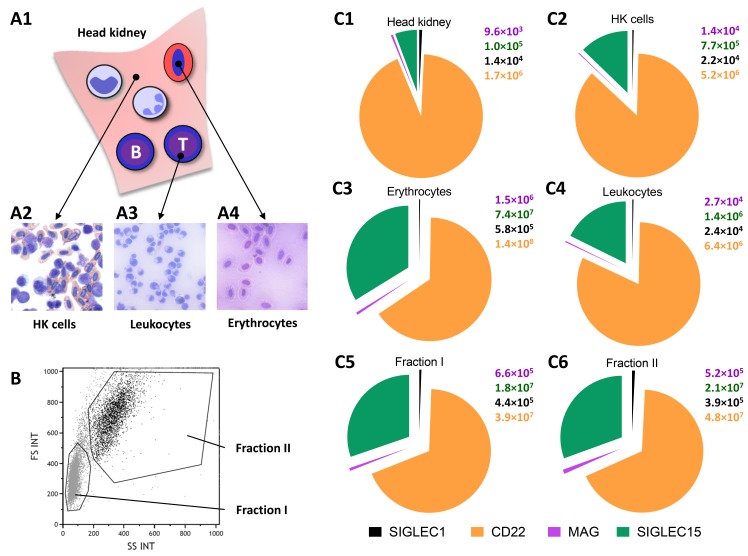
Copy numbers (per µg RNA) of *Siglec1*, *CD22*, *MAG*, and *Siglec15* in (**A1**) whole head kidneys (HKs), (**A2**) extracted HK cells, (**A3**) a heterogeneous leukocyte suspension, (**A4**) erythrocytes, and (**B**) one sorted cell fraction (I), enriched with lymphocytes and monocytes/macrophages, and one fraction (II) enriched with granulocytes (n = 5). (**C**) Siglec expression in the isolated cell fractions (**C1**) HK, (**C2**) HK cells, (**C3**) erythrocytes, (**C4**) leukocytes, (**C5**) fraction I, and (C**6**) fraction II. QPCR expression data for *Siglec1* (black), *CD22* (orange), *MAG* (purple), and *Siglec15* (green) was normalized against the GeoMean of the reference genes *RPL9*, *EEF1A1b*, and *RPL32*.

Since in mammals Siglecs are heterogeneously expressed in immune cells [39], we conducted a subsequent qPCR analysis to determine which immune-cell populations express Siglecs in maraena whitefish. To this end, we isolated the HKs (Figure 3A1) and extracted cells from these tissues (Figure 3A2). The resulting cell suspension was then separated into leukocyte (Figure 3A3) and erythrocyte (Figure 3A4) suspensions via Percoll treatment. Eventually, the leucocyte suspension was further separated into a fraction I, enriched with less granular and smaller cells (presumably lymphocytes and monocytes/macrophages), and a fraction II with more granular and larger cells (presumably granulocytes) (Figure 3B). Profiling the copy numbers of *Siglec1*, *CD22*, *MAG*, and *Siglec15* via multiplex qPCR revealed that the erythrocytes were the main cell population expressing all four Siglecs (Figure 3C1–6).

The main function of mammalian erythrocytes is the transportation of oxygen, whereas teleostean erythrocytes also exert immunological functions [40,41,42,43]. This may be due to the fact that teleostean erythrocytes contain nuclei and are capable of regulating their gene expression if necessary [44], in contrast to their mammalian counterparts [45,46]. In particular, fish erythrocytes are considered as antigen-presenting cells, recognize pathogen-associated molecular patterns (PAMPs), phagocytose, and influence the activity of other immune cells [40,41,42,43]. It is likely that Siglecs are also involved in these erythrocyte-pathogen interactions. A closer look at the Siglec expression in erythrocytes from maraena whitefish revealed that *Siglec15* and *CD22* were most highly expressed. This also applied to all other immune cell fractions indicating that a high expression of *CD22* in fish is not restricted to B cells [47]. The expression of mammalian *CD22* has been described as being predominately located in B cells [47]. As the genomes of fish obviously lack any inhibitory CD33-related Siglecs (containing the ITIM motif), it might be possible that CD22 may take over this role in the immune cells of fish. In addition, the expression of *MAG* and *Siglec1* was detectable in the analyzed immune cell populations of maraena whitefish.

### 3.3. Sequence Comparison of Siglecs Expressed by Salmonid and Percid Fishes

We analyzed the nucleotide and amino-acid sequences of the evolutionarily conserved Siglecs in fish and mammals in more detail because their expression profiles exhibited remarkable differences. Siglec1 contains no regulatory domains; for this reason, only the sequences of CD22, MAG, and Siglec15 are presented here. Since the Siglec sequences of the pikeperch (SIGLEC1L, CD22L, MAGL, and CD33L) revealed poor homology with its piscine orthologs (see Section 2.5), we disregarded these sequences in the more detailed sequence analysis.

#### 3.3.1. Sequence Comparison of Siglec2 (CD22)

In mammals, CD22 can counteract the BCR-triggered activation of B cells, when defined sialylated structures are detected simultaneously with an antigen [48,49]. The clustering of a BCR with CD22 molecules causes the recruitment of SHP1 and SHP2 (encoded by the genes *PTPN6* and *PTPN11*), which leads to the inhibition of the kinase-dependent signaling pathway, along with the reduced production of antibodies against the autoantigen [48,49]. This mechanism inhibits thus the synthesis of autoantibodies. Teleosts produce three main types of immunoglobulins (IgM, IgD, and IgT/IgZ) that act as BCRs [50,51]. The interplay between these immunoglobulins and CD22 might be regulated in a mammalian-analogous way on teleostean B cells.

**Figure 4:**
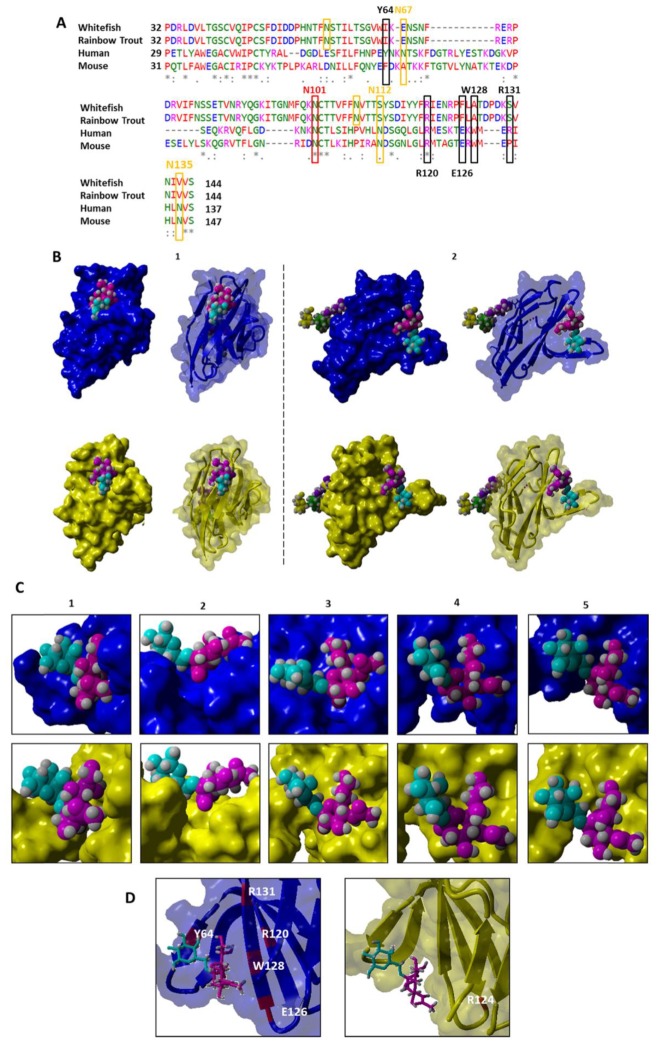
Sequence and structural comparison of CD22 from human and maraena whitefish. (**A**) The first Ig domain of maraena whitefish and rainbow trout was determined by aligning sequences. The different colors indicate the chemical properties of the amino acids as follows: red, small hydrophobic/aromatic amino acids; blue, acidic amino acids; magenta, basic amino acids; green, hydrophilic, polar, and small amino acids. Black boxes show the amino acids that are essential for sialic acid-binding; red boxes show the conserved N-glycosylation site N_101_. In addition, the orange boxes indicate further amino acids known to be a target for N-glycosylation. Numbering is based on the human CD22 sequence. (**B**) YASARA was used to model the 3D structure of the sialic-acid-binding domain (V-set, Ig-like domain) of CD22 from human (pdb: 5VKM, including 5 point mutations N_67_A, N_112_A, N_135_A, N_164_A, N_231_A [30]) and maraena whitefish. The sequence of the human MAG was shortened to the first Ig domain. The sequence of the V-set, Ig-like domain of CD22 from maraena whitefish was based on the alignments (see (**A**)). CD22 models from human and whitefish are labelled in blue and yellow, respectively. Two different perspectives of the surface with a transparency of 30% are shown along with the corresponding secondary structures. (**C**) Enlargement of the sialic-acid-binding domain of CD22 from maraena whitefish in five different perspectives. (**D**) Amino acids responsible for sialic acid-binding by CD22 from human and maraena whitefish. Surface and secondary structures are shown. Bound glycans are specifically labeled as follows: galactose, cyan; *N*-acetyl-d-galactosamine, purple; sialic acid, pink; α-D-mannose, yellow; β-D-mannose, green.

The *N*-glycosylation status of mammalian CD22 seems to be important for its activity. Twelve *N*-glycosylation sites are known in mammalian CD22, six of which are close to the sialic acid-binding domain (N_67_; N_101_; N_112_; N_135_; N_164_; N_231_). According to Orbea et al., N_67_, N_112_, N_135_, N_164_, N_231_ can be exchanged by an alanine without functional loss, while the mutation of N_101_ disrupted protein expression [30]. N-glycans at N_101_ are probably involved in the correct folding of the receptor. Regarding the sequence comparison of the V-set Ig-like domain, which is responsible for sialic acid-binding (Figure 4A), N_101_ seems to be conserved from mammals to lower vertebrates (N_105_ in fishes), while N_67_ and N_135_ seem to be absent. Recently, Wasim et al. determined that mutations of N_67_, N_112_, N_135_, N_164_ and N_231_ resulted in a higher density of CD22 nanoclusters, along with a decreased CD22-phosphorylation rate and an increased B-cell signaling, culminating in a reduced functionality of CD22 [52]. Therefore, we took a closer look at the N-glycosylation sites in the CD22 orthologs from maraena whitefish and rainbow trout. The alignment of the CD22 sequences from maraena whitefish and rainbow trout suggested (Figure 4A and Figure S1) that the majority of N-glycosylation sites are also present in CD22 of fish, although they are at slightly different positions compared to their human orthologs. The N-glycosylation at N_67_, N_101_, N_112_, N_164_ and N_231_ in human corresponds to N_59_, N_105_, N_112_, N_167_ and N_221_ in fish. Moreover, we searched for ITIM domains in the CD22 orthologs of maraena whitefish and rainbow trout, since these domains characterize inhibitory receptors in mammals [53]. An ITIM is present in CD22 of rainbow trout but not in the orthologous sequence of maraena whitefish (Figure S1). However, our CD22 sequence of maraena whitefish was severely truncated, and we cannot exclude the possibility that an ITIM is present there.

Furthermore, we analyzed the sialic acid-binding domain in more detail. Human CD22 preferentially binds α2,6-linked sialic acid. The binding is mediated by the amino-acid residues R_120_, R_131_, E_126_ and W_128_ in addition to Y_64_, which is responsible for the preference for α2,6-linked sialic acid [30]. The sequence comparison (Figure 4A) demonstrated that R_120_ is conserved from mammals to fish, while almost all other amino acids, necessary for sialic acid-binding in humans, are missing in the investigated fish. In mice, Y_64_ is replaced by F, indicating the conservation of the aromatic properties, and also in fish, W resides close to Y_64_ in human [30].

In addition, we simulated 3D models of the V-set Ig-like domain of CD22 from maraena whitefish based on the known 3D structure of the human counterpart [30]. This 3D model exhibited remarkable structural differences between the CD22 orthologs from human and maraena whitefish (Figure 4B–D). Hence, based on the modelling of CD22 from maraena whitefish (Figure 4B–D) combined with the sequence alignment (Figure 4A), we suggest that the binding properties of CD22 have changed during evolution. However, experimental data is needed to define the glycan-binding properties of CD22 in fishes.

#### 3.3.2. Sequence Comparison of Siglec15

**Figure 5:**
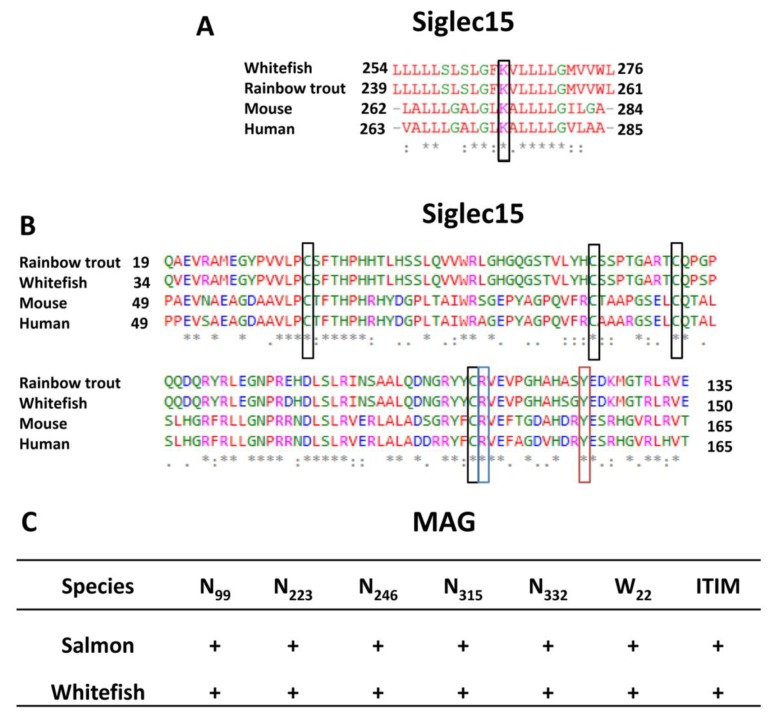
Analysis of specific properties of Siglec15 and MAG. (**A**) Sequence alignment of the transmembrane domain of Siglec15. The positions of the transmembrane domains of murine and human Siglec15 were retrieved from Uniprot, while the transmembrane area of Siglec15 from maraena whitefish and rainbow trout were predicted using SMART. The black box highlights the transmembrane lysine residue, which interacts with DAP10/12. (**B**) Sequence alignments of the first Ig domain of Siglec15. The alignment illustrates the conserved cysteine residues (black boxes) of Siglec15 as well as the conserved arginine residue (blue box) and a conserved hydrophobic amino acid (Y, red box), which are involved in sialic acid-binding [17]. Sequence alignments were performed using the Clustal Omega tool of EMBL-EBI. Different colors label the chemical properties of the amino acids: red, small hydrophobic/aromatic amino acids; blue, acidic amino acids; magenta, basic amino acids; green, hydrophilic, polar, and small amino acids. (**C**) The presence of specific N-glycosylation sites and IIM motifs of MAG from salmon and whitefish are shown. The position of the amino acid residue refers to the human sequence. For accession numbers, see Section 2.7.

Siglec15 belongs to the activating receptors, interacting with DAP10/12 via a lysine residue in the transmembrane domain [17]. This residue is well-conserved from fish to mammals (Figure 5A). The exchange of lysine by alanine has been demonstrated to abrogate the interaction of Siglec15 with DAP10 or DAP12; this confirms the functional importance of the lysine residue. However, this exchange might not impact the minimal FcRγ interaction with Siglec15 [17].

Furthermore, sequence alignments of the first Ig domain were performed. Angata and colleagues showed that an exchange of R_143_ to alanine results in the loss of sialic acid-binding and thus, in a loss of the immune regulatory function of Siglec15 [17]. This amino acid is conserved from mammals to lower vertebrates (Figure 5B). In addition, cysteine residues in the V-set, Ig-like domain are highly conserved across vertebrates. One likely reason for this remarkable conservation is pathogen-driven selection pressure. Our sequence alignments of the first Ig domain of the Siglec15 orthologs suggested that the well-conserved cysteine residues that contribute to the tertiary structure by forming disulfide bonds are present in the Siglec15 of the investigated salmonid species (Figure 5B). Nevertheless, the cloned ortholog from zebrafish did not show strong binding to the tested glycans ex vivo, irrespective of the presence or absence of cysteine residues [17].

#### 3.3.3. Sequence Comparison of Siglec4 (MAG)

In mammals, MAG is involved in myelination processes through interactions with gangliosides. The expression of *MAG* is restricted to Schwann cells and oligodendrocytes. The dimerization of MAG is essential for specific axon-myelin spacing (9–12 nm) and strongly depends on the glycosylation pattern of MAG [15,35,54,55]. Therefore, we also inspected potential N-glycosylation sites of the MAG sequences from maraena whitefish and Atlantic salmon. Eight sites of N-glycosylation are known in human MAG (N_99_, N_223_, N_246_, N_315_, N_332_, N_406_, N_450_, and N_454_). The residue W_22_ is targeted by *C*-mannosylation and conserved from fish to humans (W_21_ in fish). Therefore, it is likely that this residue contributes to the functionality of MAG [31]. Our sequence alignment showed that the N-glycosylation sites of the three Ig-domains of the MAG were conserved together with residue W_22_ of the murine MAG sequences (Figure 5C and Figure S2).

**Figure 6:**
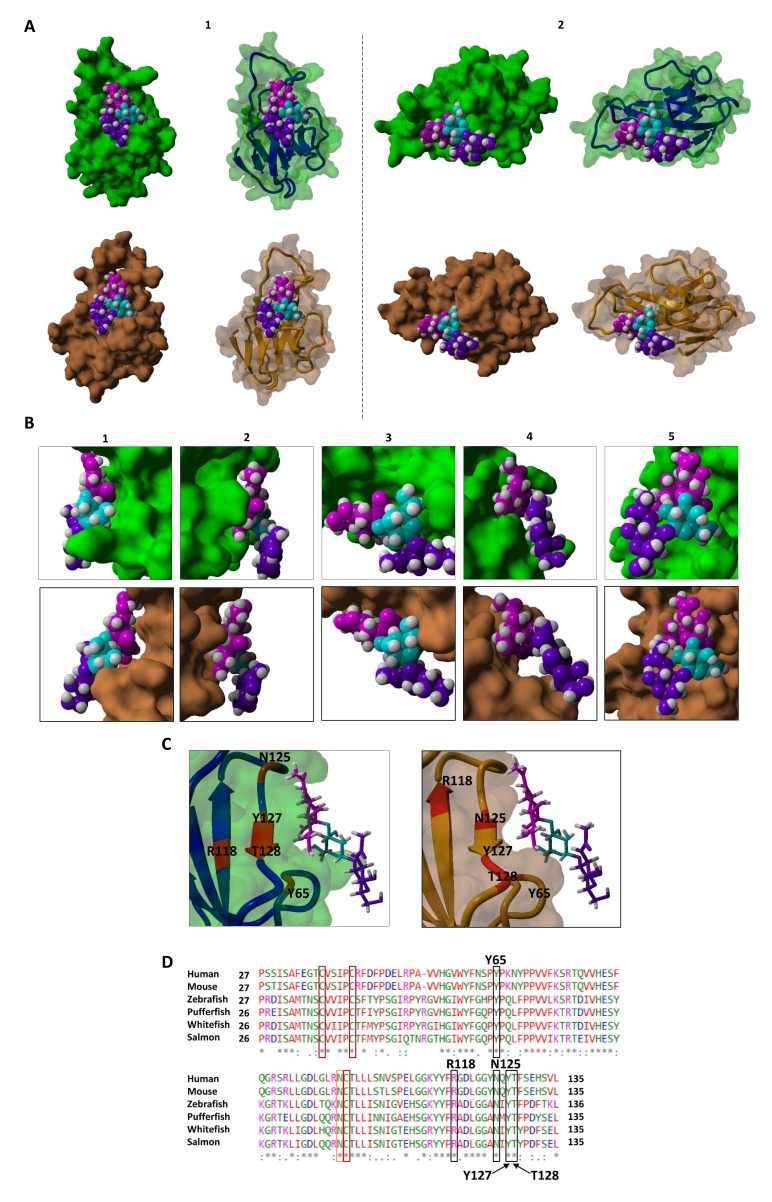
Sequence and structural comparison of MAG from mouse and maraena whitefish. (**A**) YASARA was used to model the 3D structure of the sialic-acid-binding domain (V-set, Ig-like domain) of MAG from mouse and maraena whitefish. Based on the sequence alignments performed, the sequence of the murine MAG was shortened to the first Ig domain. The sequence of the V-set Ig-like domain of MAG from maraena whitefish was based on the performed alignments (see (**D**)). MAG models from mouse and maraena whitefish are labelled in green and brown, respectively. Two different perspectives of the surface with a transparency of 30% are shown along with the corresponding secondary structures. (**B**) Enlargement of the sialic-acid-binding domain of MAG from maraena whitefish in five different perspectives. (**C**) Amino acids responsible for sialic acid-binding by MAG from mouse and maraena whitefish. Surface and secondary structures are shown. Bound glycans are specifically labeled as follows: galactose, cyan; *N*-acetyl-d-galactosamine, purple; sialic acid, pink. (**D**) Sequences of MAG from zebrafish, pufferfish, human, and mouse were available in the NCBI database: zebrafish MAG: XP_021337068; pufferfish MAG: XP_011616490; murine MAG: XP_030098048; human MAG: AAB58805. The V-set, Ig-like domain of the mouse MAG was determined by analyzing the pdb sequence (pdb: 5LF5) with SMART. The V-set Ig-like domain of the human MAG was assessed using SMART. For zebrafish, pufferfish, maraena whitefish, and rainbow trout, V-set, Ig-like domains were detected using sequence alignment. The different colors indicate the chemical properties of the amino acids as follows: red, small hydrophobic/aromatic amino acids; blue, acidic amino acids; magenta, basic amino acids; green: hydrophilic, polar, and small amino acids. Black boxes show the amino acids that are essential for sialic acid-binding, red boxes show conserved cysteine residues [16] and the orange box indicates a glycosylation site.

In addition, these MAG sequences harbor immune-regulatory ITIMs in their intracellular sections, whereas their mammalian counterparts lack these motifs. In 2004, Lehmann et al. detected a proximal ITIM motif in the MAG orthologs of zebrafish and pufferfish [16]. Lehmann et al. suggested that these motifs are involved in signal transduction and contribute to biological functions different from those described for mammalian MAG [16]. Furthermore, our previous sequence analysis [15] confirmed the presence of ITIMs in coelacanth, while almost all investigated higher vertebrates were ITIM-negative. Consequently, the ITIM motif seems to be an ancestral feature that has persisted in most Siglecs but was lost during the evolution of higher vertebrates [15,16]. The presence of ITIM motifs in the intracellular part of MAG in salmonid fish might contribute to inhibiting immunological responses, while mammalian MAGs merely stabilize axon-myelin interactions by binding to GD1a and GT1b, two gangliosides present in the brain. Unlike the MAG in mammals, the MAG in fish seems to be involved in immunomodulatory processes. To analyze its carbohydrate-binding pocket in more detail, the 3D structure of MAG from maraena whitefish was simulated and compared with its murine ortholog (Figure 6A–C).

Based on the crystal structure of murine MAG, the program determined that the secondary structure of MAG from maraena whitefish contains 37.6% beta sheets, 18.3% alpha-turn-helices, and 44.0% coiled coils, whereas that of murine MAG contains only 34.9% beta sheets, 22.0% alpha-turn-helices, and 43.1% coiled coils (Figure 6A). Although several amino acid residues differed between the MAG sequences from mouse and maraena whitefish, no significant changes were visible with regard to the sialic-acid-binding domain (Figure 6B,C). The binding pockets of MAG from mouse and maraena whitefish for Neu5Ac-α2,3-Gal-β1,3-GalNAc are highly comparable. The data suggest analogous functions of MAG in maraena whitefish and mammals. In addition, we aligned the sequences of the selected MAG orthologs to compare the presence of essential amino acids that mediate the protein-carbohydrate interaction. In human MAG, amino acids R_118_, Y_65_, N_125_, T_128_, and Y_127_ are responsible for sialic acid-binding [31]. These amino acids were conserved throughout evolution (Figure 6D), indicating once more [16] the preserved potential of MAG to bind to sialic acid across a range of vertebrate classes.

### 3.4. The Influence of Handling Stress on the Expression of Siglecs in Maraena Whitefish

**Figure 7:**
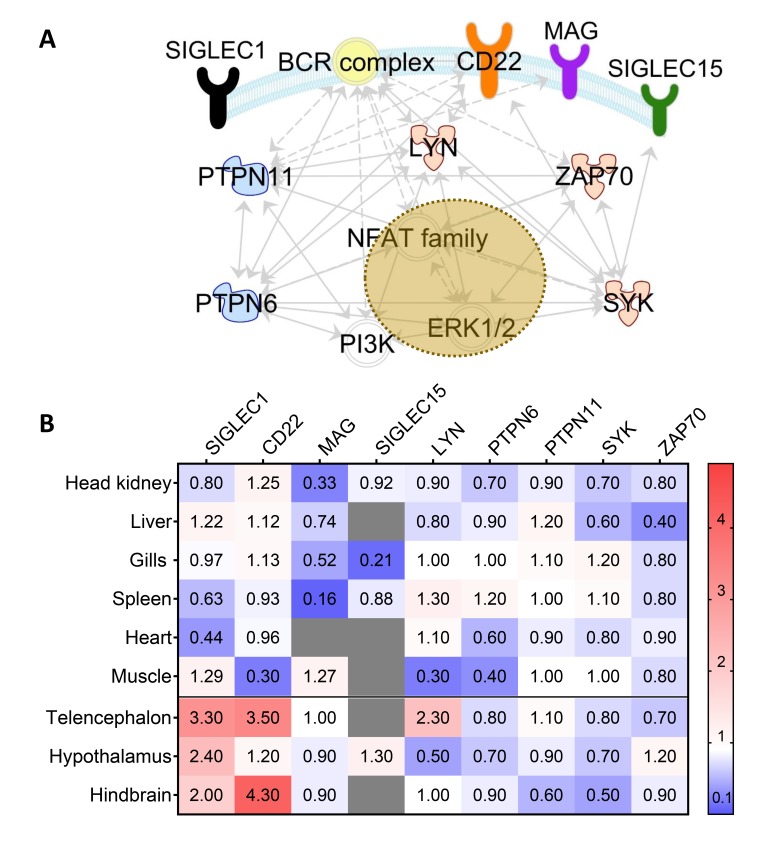
(**A**) Functional relationship between Siglec receptors and associated kinases (red symbols) and phosphatases (blue symbols) in a schematic B cell. Interactions are displayed by broken (indirect influence) or full (direct influence) lines. Cellular and nuclear membranes are colored in light blue and brown, respectively. Please note that these relationships are supported by at least one reference, which are based exclusively on investigations in mammalian species (accessible in the Ingenuity Knowledge Base). (**B**) Tissue-specific expression of genes encoding Siglecs (*Siglec1*, *CD22*, *MAG*, *Siglec15*) and downstream factors (*LYN*, *PTPN6*, *PTPN11*, *SYK*, *ZAP70*) in maraena whitefish exposed to three hours of handling stress. QPCR data were normalized by the reference genes *RPL9*, *EEF1A1b*, and *RPL32*. The heat map shows the averaged fold-change values in the respective tissue relative to the same tissue from unstressed fish, colored according to the code on the right. Non-detectable transcript numbers are indicated by gray fields.

Stress is known to affect immune processes [2]. To investigate the impact of stress on a panel of nine selected target genes related to the Siglec signaling, we exposed maraena whitefish to one-minute handling procedures (including chasing and exposure to air) and sampled the fish three hours after this treatment. Subsequently, we recorded the expression of the genes encoding the four Siglecs present in fish (*Siglec1*, *CD22*, *MAG*, and *Siglec15*), the associated non-receptor tyrosine kinases *LYN, SYK,* and *ZAP70* in addition to the non-receptor tyrosine phosphatase *PTPN6* (alias SHP1) and *PTPN11* (alias SHP2) (Figure 7A). Gene profiling revealed that the transcript levels of the four Siglecs were modulated in a tissue-specific fashion after exposure to stress (Figure 7B). *Siglec1* and *CD22* were 2.0- to 4.3-fold upregulated in telencephalon and hindbrain but substantially downregulated in the heart as well as the spleen (*Siglec1*) and muscle (*CD22*). All other tissues exhibited comparable values in untreated and stressed fish.

Surprisingly, *MAG* expression was not influenced in the brain and was downregulated in nearly all other tissues. This effect was especially pronounced in both lymphoid organs spleen and HK. Since MAG from maraena whitefish contains an ITIM, this data might indicate the immunomodulatory capacity of MAG in salmonid fish. Furthermore, few *MAG* transcripts were detected in gills of stressed maraena whitefish, and these fish also exhibited reduced *Siglec15*-transcript level in their gills. As the respiratory organs of fish, gills are directly exposed to significant environmental changes, including exposure to air, and are thus expected to induce fast responses.

The genes encoding the three Siglec-associated kinases (*LYN, SYK,* and *ZAP70*) and two phosphatases (*PTPN6* and *PTPN11*) were expressed at high levels (between ~2200 and ~71,500 copies/µg RNA) in HK, gills and spleen, but at low levels (<450 copies/µg RNA) in muscle, telencephalon, hypothalamus, and hindbrain. Handling stress did not affect the expression of the aforementioned enzyme genes, except for *LYN* in muscle (~4-fold downregulated), hypothalamus (~2-fold downregulated), and telencephalon (~2-fold upregulated), as well as *PTPN6* in muscle (~3-fold downregulated) (Figure 7B). Since we recorded these alterations in those tissues that had only relatively low basal concentrations of the respective transcripts, the observed expression data should not be overestimated. We rather assume that the stress-dependent regulation of the activity of Siglec-associated enzymes in fish does not occur at the transcript level. In contrast, the expression patterns of Siglec-encoding genes showed characteristic factor-specific alterations, both under homeostatic conditions and in response to handling stress. Although the biological significance of these changes remains unknown, the data obtained in the present study strongly suggests that the function of individual Siglecs has partially changed during the evolution of vertebrates.

## 4. Conclusions

The present study draws three main conclusions:

- Our qPCR analyses suggested that the basal gene-expression patterns of *Siglec1*, *CD22*, *MAG*, and *Siglec15* are largely conserved across salmonid and percid fishes. In contrast to mammals, *CD22* is highly expressed in several blood-cell populations. Similarly, the expression of *MAG* in fish is not restricted to the cells of the nervous system but is detectable in a range of blood cells.
- Stress modulates the expression of Siglecs (but not of the associated enzymes) in a tissue-dependent fashion and most likely influences the cellular reactivity against PAMPs and DAMPs.
- The genomes of fish lack CD33-related Siglecs, which exert inhibitory functions. Our structural analyses indicated that CD22 and MAG contain inhibitory motifs (ITIM) in salmonid fish. We speculate that these ITIM-containing Siglecs may compensate the deficiency of the canonical inhibitory Siglecs. This first assumption might be the starting point for subsequent studies to clarify whether CD22 and MAG have an immunosuppressive effect in fish.

## Supplementary Materials

The following materials are available online at https://www.mdpi.com/2073-4409/9/4/836/s1: Figure S1: Sequence alignment of CD22 of rainbow trout (XM_021620093) and the obtained sequence of CD22 of maraena whitefish, Figure S2: Sequence alignment of MAG from salmon and the obtained sequence of MAG of maraena whitefish, Table S1: Primer sequences and accession codes.

## References

[1] Uribe C., Folch H., Enríquez R., Moran G. (2011). Innate and adaptive immunity in teleost fish: A review. Vet. Med..

[2] Rebl A., Goldammer T. (2018). Under control: The innate immunity of fish from the inhibitors’ perspective. Fish Shellfish Immunol..

[3] Varki A. (2017). Are humans prone to autoimmunity? Implications from evolutionary changes in hominin sialic acid biology. J. Autoimmun..

[4] Varki A. (2017). Biological roles of glycans. Glycobiology.

[5] Varki A. (2011). Letter to the glyco-forum: Since there are pamps and damps, there must be samps? Glycan “self-associated molecular patterns” dampen innate immunity, but pathogens can mimic them. Glycobiology.

[6] Bloemendal V.R.L.J., Moons S.J., Heming J.J.A., Chayoua M., Niesink O., van Hest J.C.M., Boltje T.J., Rutjes F.P.J.T. (2019). Chemoenzymatic synthesis of sialic acid derivatives using immobilized n-acetylneuraminate lyase in a continuous flow reactor. Adv. Synth. Catal..

[7] Coady A., Nizet V. (2016). Samp-ending down sepsis. Ann. Transl. Med..

[8] Varki A., Angata T. (2006). Siglecs—The major subfamily of i-type lectins. Glycobiology.

[9] Crocker P.R., Paulson J.C., Varki A. (2007). Siglecs and their roles in the immune system. Nat. Rev. Immunol..

[10] Angata T., Margulies E.H., Green E.D., Varki A. (2004). Large-scale sequencing of the cd33-related siglec gene cluster in five mammalian species reveals rapid evolution by multiple mechanisms. Proc. Natl. Acad. Sci. USA.

[11] Ravetch J.V., Lanier L.L. (2000). Immune inhibitory receptors. Science.

[12] Pillai S., Netravali I.A., Cariappa A., Mattoo H. (2012). Siglecs and immune regulation. Annu. Rev. Immunol..

[13] O’Reilly M.K., Paulson J.C. (2009). Siglecs as targets for therapy in immune-cell-mediated disease. Trends Pharmacol. Sci..

[14] Crocker P.R., Varki A. (2001). Siglecs in the immune system. Immunology.

[15] Bornhöfft K.F., Goldammer T., Rebl A., Galuska S.P. (2018). Siglecs: A journey through the evolution of sialic acid-binding immunoglobulin-type lectins. Dev. Comp. Immunol..

[16] Lehmann F., Gathje H., Kelm S., Dietz F. (2004). Evolution of sialic acid-binding proteins: Molecular cloning and expression of fish siglec-4. Glycobiology.

[17] Angata T., Tabuchi Y., Nakamura K., Nakamura M. (2007). Siglec-15: An immune system siglec conserved throughout vertebrate evolution. Glycobiology.

[18] Robertson F.M., Gundappa M.K., Grammes F., Hvidsten T.R., Redmond A.K., Lien S., Martin S.A.M., Holland P.W.H., Sandve S.R., Macqueen D.J. (2017). Lineage-specific rediploidization is a mechanism to explain time-lags between genome duplication and evolutionary diversification. Genome Biol..

[19] Korytar T., Nipkow M., Altmann S., Goldammer T., Kollner B., Rebl A. (2016). Adverse husbandry of maraena whitefish directs the immune system to increase mobilization of myeloid cells and proinflammatory responses. Front. Immunol..

[20] Rebl A., Verleih M., Nipkow M., Altmann S., Bochert R., Goldammer T. (2018). Gradual and acute temperature rise induces crossing endocrine, metabolic, and immunological pathways in maraena whitefish (coregonus maraena). Front. Genet..

[21] Swirplies F., Wuertz S., Baßmann B., Orban A., Schäfer N., Brunner R.M., Hadlich F., Goldammer T., Rebl A. (2019). Identification of molecular stress indicators in pikeperch sander lucioperca correlating with rising water temperatures. Aquaculture.

[22] Rebl A., Rebl H., Verleih M., Haupt S., Kobis J.M., Goldammer T., Seyfert H.M. (2019). At least two genes encode many variants of irak3 in rainbow trout, but neither the full-length factor nor its variants interfere directly with the tlr-mediated stimulation of inflammation. Front. Immunol..

[23] Martorell Ribera J., Nipkow M., Viergutz T., Brunner R.M., Bochert R., Koll R., Goldammer T., Gimsa U., Rebl A. (2020). Early response of salmonid head-kidney cells to stress hormones and toll-like receptor ligands. Fish Shellfish Immunol..

[24] Nguinkal J.A., Brunner R.M., Verleih M., Rebl A., de Los Rios-Perez L., Schafer N., Hadlich F., Stueken M., Wittenburg D., Goldammer T. (2019). The first highly contiguous genome assembly of pikeperch (sander lucioperca), an emerging aquaculture species in europe. Genes (Basel).

[25] Langmead B., Salzberg S.L. (2012). Fast gapped-read alignment with bowtie 2. Nat. Methods.

[26] Altmann S., Rebl A., Kuhn C., Goldammer T. (2015). Identification and de novo sequencing of housekeeping genes appropriate for gene expression analyses in farmed maraena whitefish (coregonus maraena) during crowding stress. Fish Physiol. Biochem..

[27] Rebl A., Rebl H., Korytar T., Goldammer T., Seyfert H.M. (2014). The proximal promoter of a novel interleukin-8-encoding gene in rainbow trout (oncorhynchus mykiss) is strongly induced by cebpa, but not nf-kappab p65. Dev. Comp. Immunol..

[28] Rebl A., Anders E., Wimmers K., Goldammer T. (2009). Characterization of dehydrodolichyl diphosphate synthase gene in rainbow trout (oncorhynchus mykiss). Comp. Biochem. Physiol. B Biochem. Mol. Biol..

[29] Brietzke A., Borchel A., Altmann S., Nipkow M., Rebl A., Brunner R.M., Goldammer T. (2016). Transcriptome sequencing of maraena whitefish (coregonus maraena). Mar. Genomics.

[30] Ereño-Orbea J., Sicard T., Cui H., Mazhab-Jafari M.T., Benlekbir S., Guarné A., Rubinstein J.L., Julien J.P. (2017). Molecular basis of human cd22 function and therapeutic targeting. Nat. Commun..

[31] Pronker M.F., Lemstra S., Snijder J., Heck A.J.R., Thies-Weesie D.M.E., Pasterkamp R.J., Janssen B.J.C. (2016). Structural basis of myelin-associated glycoprotein adhesion and signalling. Nat. Commun..

[32] Chang Y.C., Olson J., Louie A., Crocker P.R., Varki A., Nizet V. (2014). Role of macrophage sialoadhesin in host defense against the sialylated pathogen group b streptococcus. J. Mol. Med. (Berl)..

[33] Zapata A., Diez B., Cejalvo T., Gutierrez-de Frias C., Cortes A. (2006). Ontogeny of the immune system of fish. Fish Shellfish Immunol..

[34] Macauley M.S., Crocker P.R., Paulson J.C. (2014). Siglec regulation of immune cell function in disease. Nat. Rev. Immunol..

[35] Quarles R.H. (2007). Myelin-associated glycoprotein (mag): Past, present and beyond. J. Neurochem..

[36] Kopatz J., Beutner C., Welle K., Bodea L.G., Reinhardt J., Claude J., Linnartz-Gerlach B., Neumann H. (2013). Siglec-h on activated microglia for recognition and engulfment of glioma cells. Glia.

[37] Blasius A.L., Colonna M. (2006). Sampling and signaling in plasmacytoid dendritic cells: The potential roles of siglec-h. Trends Immunol..

[38] Mott R.T., Ait-Ghezala G., Town T., Mori T., Vendrame M., Zeng J., Ehrhart J., Mullan M., Tan J. (2004). Neuronal expression of cd22: Novel mechanism for inhibiting microglial proinflammatory cytokine production. Glia.

[39] Duan S., Paulson J.C. (2020). Siglecs as immune cell checkpoints in disease. Annu. Rev. Immunol..

[40] Puente-Marin S., Nombela I., Ciordia S., Mena M.C., Chico V., Coll J., Ortega-Villaizan M.D.M. (2018). In silico functional networks identified in fish nucleated red blood cells by means of transcriptomic and proteomic profiling. Genes (Basel).

[41] Passantino L., Massaro M.A., Jirillo F., Di Modugno D., Ribaud M.R., Modugno G.D., Passantino G.F., Jirillo E. (2007). Antigenically activated avian erythrocytes release cytokine-like factors: A conserved phylogenetic function discovered in fish. Immunopharmacol. Immunotoxicol..

[42] Morera D., Roher N., Ribas L., Balasch J.C., Donate C., Callol A., Boltana S., Roberts S., Goetz G., Goetz F.W. (2011). Rna-seq reveals an integrated immune response in nucleated erythrocytes. PLoS ONE.

[43] Workenhe S.T., Kibenge M.J., Wright G.M., Wadowska D.W., Groman D.B., Kibenge F.S. (2008). Infectious salmon anaemia virus replication and induction of alpha interferon in atlantic salmon erythrocytes. Virol. J..

[44] Puente-Marin S., Thwaite R., Mercado L., Coll J., Roher N., Ortega-Villaizan M.D.M. (2019). Fish red blood cells modulate immune genes in response to bacterial inclusion bodies made of tnfα and a g-vhsv fragment. Front. Immunol..

[45] Raja-Sabudin R.Z., Othman A., Ahmed-Mohamed K.A., Ithnin A., Alauddin H., Alias H., Abdul-Latif Z., Das S., Abdul-Wahid F.S., Hussin N.H. (2014). Immature reticulocyte fraction is an early predictor of bone marrow recovery post chemotherapy in patients with acute leukemia. Saudi Med. J..

[46] Ney P.A. (2011). Normal and disordered reticulocyte maturation. Curr. Opin. Hematol..

[47] Clark E.A., Giltiay N.V. (2018). Cd22: A regulator of innate and adaptive b cell responses and autoimmunity. Front. Immunol..

[48] Nitschke L. (2005). The role of cd22 and other inhibitory co-receptors in b-cell activation. Curr. Opin. Immunol..

[49] Tedder T.F., Poe J.C., Haas K.M. (2005). Cd22: A multifunctional receptor that regulates B lymphocyte survival and signal transduction. Adv. Immunol..

[50] Parra D., Korytář T., Takizawa F., Sunyer J.O. (2016). B cells and their role in the teleost gut. Dev. Comp. Immunol..

[51] Parra D., Takizawa F., Sunyer J.O. (2013). Evolution of b cell immunity. Annu. Rev. Anim. Biosci..

[52] Wasim L., Buhari F.H.M., Yoganathan M., Sicard T., Ereño-Orbea J., Julien J.-P., Treanor B. (2019). N-linked glycosylation regulates cd22 organization and function. Front. Immunol..

[53] Muller J., Obermeier I., Wohner M., Brandl C., Mrotzek S., Angermuller S., Maity P.C., Reth M., Nitschke L. (2013). Cd22 ligand-binding and signaling domains reciprocally regulate B-cell Ca^2+^ signaling. Proc. Natl. Acad. Sci. USA.

[54] Sun J., Shaper N.L., Itonori S., Heffer-Lauc M., Sheikh K.A., Schnaar R.L. (2004). Myelin-associated glycoprotein (siglec-4) expression is progressively and selectively decreased in the brains of mice lacking complex gangliosides. Glycobiology.

[55] Cagnoni A.J., Pérez Sáez J.M., Rabinovich G.A., Mariño K.V. (2016). Turning-off signaling by siglecs, selectins, and galectins: Chemical inhibition of glycan-dependent interactions in cancer. Front. Oncol..

