# Supplementary material for: Characterization of Sialic Acid-Binding Immunoglobulin-Type Lectins in Fish Reveals Teleost-Specific Structures and Expression Patterns
Source: Cells. 2020 Mar 31;9(4):836. doi: 10.3390/cells9040836 (PMC7226832; doi:10.3390/cells9040836)
Supplement: Supplementary file 1 [file cells-09-00836-s001.zip › Figure S1.pdf]

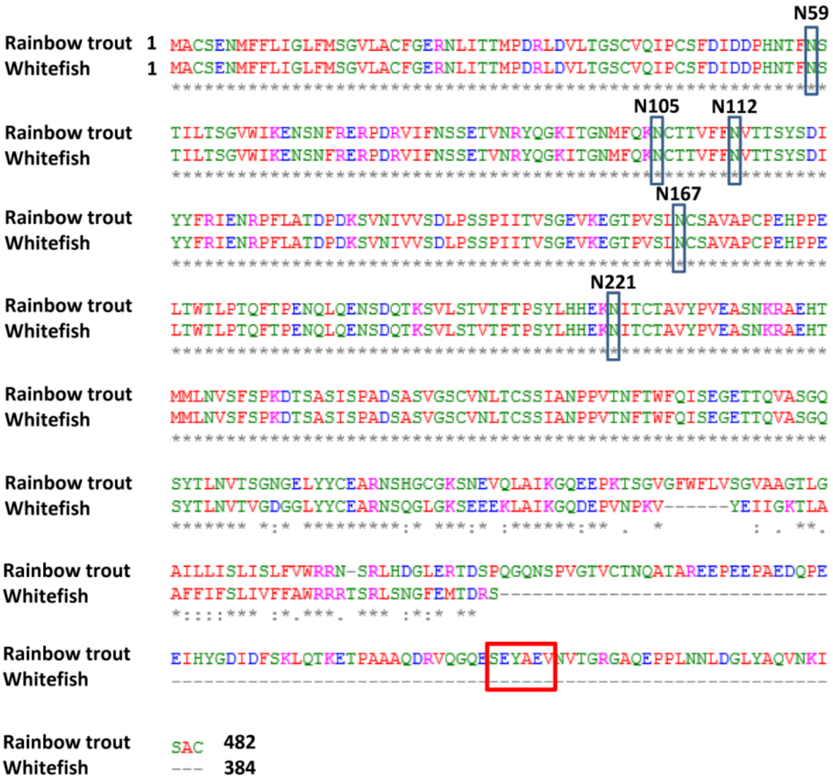

Figure S1. Sequence alignment of CD22 of rainbow trout (XM_021620093) and the obtained sequence of CD22 of maraena whitefish. Alignments were performed using the Clustal Omega tool of EMBL-EBI. The different colors indicate the properties of the amino acids. Red, small hydrophobic/aromatic amino acids; blue, acidic amino acids; magenta, basic amino acids; green: hydrophil, polar and small amino acids. The red box indicates the ITIM motif: SEYAEV.
