# Supplementary material for: Characterization of Sialic Acid-Binding Immunoglobulin-Type Lectins in Fish Reveals Teleost-Specific Structures and Expression Patterns
Source: Cells. 2020 Mar 31;9(4):836. doi: 10.3390/cells9040836 (PMC7226832; doi:10.3390/cells9040836)
Supplement: Supplementary file 1 [file cells-09-00836-s001.zip › Table S1.pdf]

Table S1. Primer sequences and accession codes.

**Official gene name (alias); gene product**	**Organ-ism**[a]	**NCBI acc. no. of seq. used for annotation**[a]	**Sense, antisense primer (5′→3′)**
SIGLEC1 (sialoadhesin); sialic acid-binding Ig-like lectin 1	*Cm*	XM_021564472 (*Om*)	ACACCTGGTACAAGAAGAAGTAAT, GCTTTTGTTGAGGCTACAGTCTT
	Om	XM_021564472 (*Om*)	CCTCCGTGTTTGTAAATGTTCAG, CTTTTGGTGAGATGACAGTCTTTT
	Sl	XM_028564630 (*Pf*)	CTCTCCTTCAGTGTGTGTCCAA, CACGACGGAACCAGAGTTTTGA
CD22 (SIGLEC2); sialic acid-binding Ig-like lectin 2	*Cm*	XM_021620093 (*Om*)	TTTATTACAGATTTGCCTCCCAGT, GGATTCTCCTGCAGTTGGTTCT
	Om	FR912501 (*Om*)	GACGAGATTTGAATGCCACAATG, GGGTTTTCTTTAATCCACACTCC
	Sl	XM_028581436 (*Pf*)	GAATGTAGAACATCCTGCCAACT, AACGGGGGATACTGAACATCCA
MAG (SIGLEC4); myelin-associated glycoprotein	*Cm*	XM_014200664 (*Ss*)	CACTGTGGAATACGCCCCTGT, TGTAGTGGCCTTCCGTGTCGT
	Om	XM_014200664 (*Ss*)	MAGa: GTCCGGTATCCGGCCACACC, AGCAGGGTGCAGTTCCTTTGTT<br>MAGb: AGTATGCCCCTCGCTCGGTGT, AGCTGCTCCTGGTCCCCAAAT
	Sl	XM_028580080 (*Pf*)	ACTGTGACTTTTATCCAAAGATCC, TGCTGTTGACTGTGTGGTGTGA
SIGLEC15 (CD33L3); sialic acid-binding Ig-like lectin 15	*Cm*	XM_021618058 (*Om*)	AGTGGAAGCTGCCCCACGGAT, GGAGAGGGGAGAGATGTCGG
	Om	XM_021618058 (*Om*)	CCTCCGTGTTTGTAAATGTTCAG, CTTTTGGTGAGATGACAGTCTTTT
	Sl	MF377634 (*Of*)	AGATTGGAGGGCCGATGTTGTA, TTTCCAAGACCTGCGTGCTATC
LYN; tyrosine-protein kinase type Lyn	*Cm*	XM_021613293 (*Om*)	TTGAAGAGTAAGGTGAATGGTGG, GTGCCAAAAGTTGTCTCTCTGC

PTPN6 (SHP1); tyrosine-protein phosphatase non-receptor type 6	*Cm*	XM_031786056 (*Ok*)	ACGAGGGAGACAAGAAGACCAA, GTCTCATCAAAGGGAAGGATGTT
PTPN11 (SHP2); tyrosine-protein phosphatase non-receptor type 11	*Cm*	XM_021602892 (*Om*)	CTATACAGCCGCAAGGAGGGA, CAACTCCGGCATGATGTCCGTT
SYK; spleen-associated tyrosine kinase	*Cm*	XM_021601579 (*Om*)	CACAGAGGTGTACGAGAGTCC, CGGCTTCTCTGTCTTCCTCATT
ZAP70; zeta chain of T-cell receptor-associated protein kinase 70	*Cm*	XM_014169597 (*Ss*)	CTATTACAAGGCACGCACAGCA, CTTGTATGGTTTTCCTCCGTAAG

[a] *Coregonus maraena* (Cm); *Oncorhynchus kisutch* (Ok); *Oncorhynchus mykiss* (Om); *Oplegnathus fasciatus* (Of); *Perca flavescens* (Pf); *Salmo salar* (Ss); *Sander lucioperca* (Sl).
