# Supplementary material for: Characterization of Sialic Acid-Binding Immunoglobulin-Type Lectins in Fish Reveals Teleost-Specific Structures and Expression Patterns
Source: Cells. 2020 Mar 31;9(4):836. doi: 10.3390/cells9040836 (PMC7226832; doi:10.3390/cells9040836)
Supplement: Supplementary file 1 [file Figure S2.pdf › Figure S2.pdf]

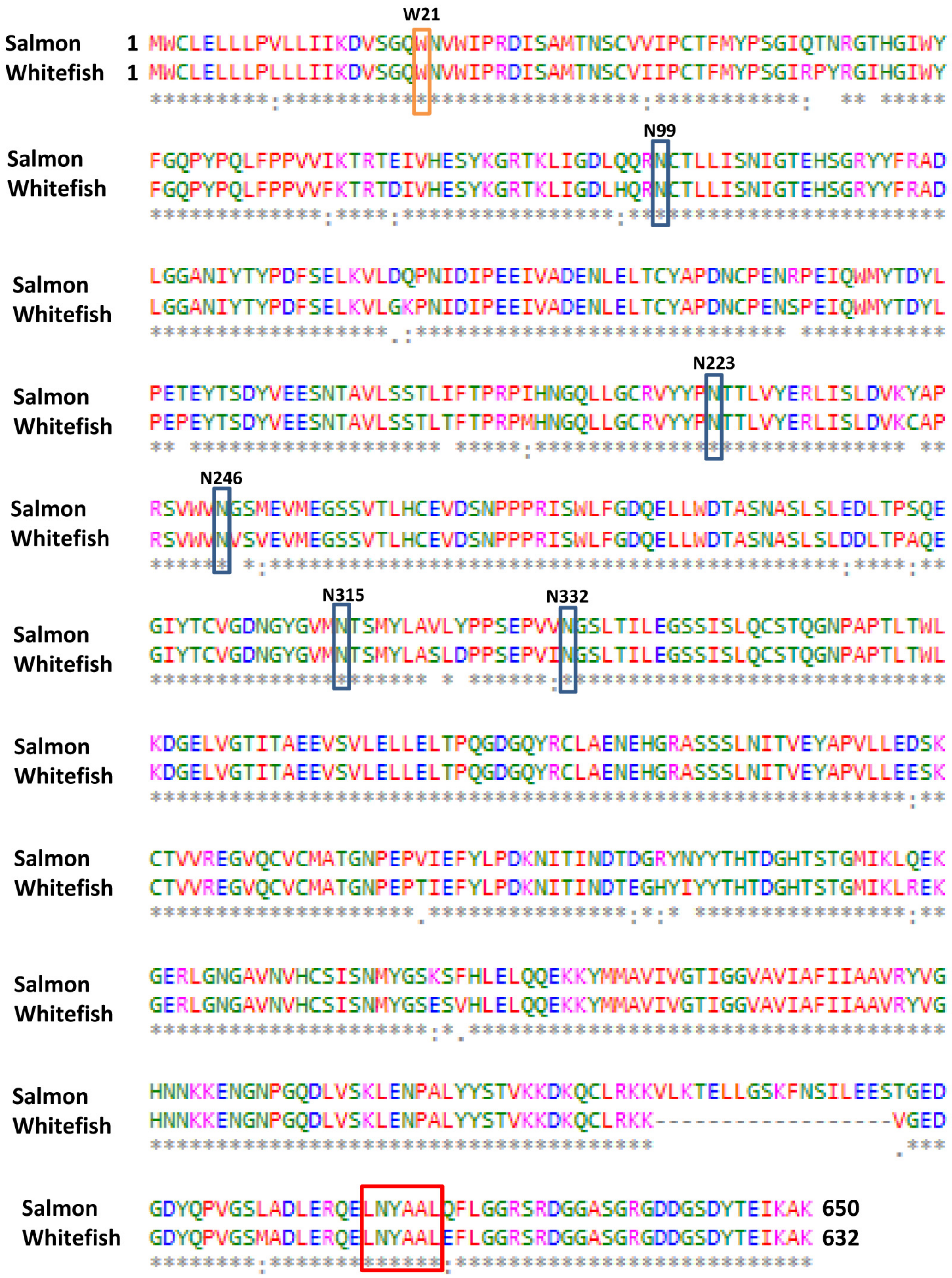

Figure S2. Sequence alignment of MAG from salmon and the obtained sequence of MAG of maraena whitefish. Alignments were performed using the Clustal Omega tool of EMBL-EBI. The different colours indicate the properties of the amino acids. Red, small hydrophobic/aromatic amino acids; blue, acidic amino acids; magenta, basic amino acids; green: hydrophilic, polar and small amino acids. The red box indicates the ITIM motif: LNYAAL, blue boxes indicate N-glycosylation sites and the orange box shows the conserved W22 (W_{21} in fish).
